# Investigation on Wireless Link for Medical Telemetry Including Impedance Matching of Implanted Antennas

**DOI:** 10.3390/s21041431

**Published:** 2021-02-18

**Authors:** Ilkyu Kim, Sun-Gyu Lee, Yong-Hyun Nam, Jeong-Hae Lee

**Affiliations:** 1 C4I Team, Defense Agency Technology and Quality, Jinju 52851, Korea; ilkyukim7@naver.com; 2 Department of Electronics and Electrical Engineering, Hongik University, Seoul 04066, Korea; gyul0206@gmail.com (S.-G.L.); namy129@naver.com (Y.-H.N.)

**Keywords:** biomedical devices, wireless communication link, near-field region, impedance matching characteristics

## Abstract

The development of biomedical devices benefits patients by offering real-time healthcare. In particular, pacemakers have gained a great deal of attention because they offer opportunities for monitoring the patient’s vitals and biological statics in real time. One of the important factors in realizing real-time body-centric sensing is to establish a robust wireless communication link among the medical devices. In this paper, radio transmission and the optimal characteristics for impedance matching the medical telemetry of an implant are investigated. For radio transmission, an integral coupling formula based on 3D vector far-field patterns was firstly applied to compute the antenna coupling between two antennas placed inside and outside of the body. The formula provides the capability for computing the antenna coupling in the near-field and far-field region. In order to include the effects of human implantation, the far-field pattern was characterized taking into account a sphere enclosing an antenna made of human tissue. Furthermore, the characteristics of impedance matching inside the human body were studied by means of inherent wave impedances of electrical and magnetic dipoles. Here, we demonstrate that the implantation of a magnetic dipole is advantageous because it provides similar impedance characteristics to those of the human body.

## 1. Introduction

Recent advancements have been made in fields related to the development of biomedical devices for the diagnosis and treatment of patients. In particular, pacemakers implanted in the chest offer real-time health care for patients who suffer from cardiac disease. Pacemakers provide the important capability of sensing intrinsic cardiac activity and transferring the pacemaker data using a wireless communication link. A class of antennas with different characteristics in terms of sizes and radiation patterns have been studied for viable in-body, on-body and off-body medical telemetries [1,2]. In addition to the antenna designs, studies on radio transmission have been performed with the aim to create reliable communication links between devices outside and inside the human body. Wireless power transfer (WPT) using electromagnetic waves can be classified into the following groups: (1) non-radiative, (2) radiative and non-radiative mid-field, and (3) radiative far-field [3]. In addition, WPT using an acoustic link can be classified as a non-EM (Electromagnetics) solution [4]. Active research has been taking place on the use of inductive coupling for non-radiative wireless links [5,6,7]. Inductive coupling has been studied with the aim to achieve a higher transfer efficiency by utilizing a hybrid microstrip and coil [5], in addition to resonators inside and outside body [6]. The design guidelines for inductive power transfer have been studied in terms of miniaturization, power consumption, and links of biomedical devices [7]. The antennas and coils used need to be interpreted as a circuit model, which requires an additional step to compute the wireless link. Radiative WPT has garnered a lot of attention due to its robustness against the misalignment of two antennas. Radio transmission of the different in-body, on-body and off-body telemetries has been investigated both by using numerical methods, such as the FDTD method, and via experimentation [8]. RFID (Radio Frequency Identification) technology for biomedical implants has been studied in terms of the wireless transfer of power and data [9]. The challenge is to calculate the wireless communication link with a reduced computational intricacy. In addition, although there have been a wide range of studies on radiative mid-field WPT [10,11], little attention has been directed towards the inclusion of accurate radiation characteristics in the near-field region. The antenna gain typically reduces as one antenna is located in the near-field region of the other antenna, which needs to be considered in the calculation of the wireless link. A simple quadratic correction term [12,13,14] to the Friis formula has been studied to increase the accuracy of the prediction in the Fresnel region. The transmission integral [15] with the plane–wave scattering matrix (PWSM) has facilitated the development of the near-field antenna measurement technique for various antennas. Based on the transmission integral, an integral form of coupling formula has been developed in order to provide the convenience of using an easily attainable far-field pattern [16]. The formula provides an enormous capacity to compute the near-field or far-field antenna coupling, which is not restricted to the type of antenna or the motion of the antenna [16,17,18,19,20]. Advancements in the formula have been made in terms of its widespread applicability to electromagnetic problems. It has been proven that the employment of a larger solid angle is beneficial for achieving converged results [19]. For microwave applications, it has been used to compute the coupling, including the dielectric structure between two antennas [19], and to provide near-field power densities of the array antenna [20]. The qualitative comparison among different techniques is summarized in Table 1. Another important aspect in realizing a reliable wireless link is the use of an optimal antenna to provide the best impedance matching in the environment of a human implant. In order to provide the best match, antennas must be electromagnetically characterized in the environment, for example, inside and outside the human body. In order to achieve improved matching characteristics, the design of a pyramidal horn antenna, including a composite material which is similar to the human skin, has been optimized [21]. The impedance characteristics of electrical and magnetic dipole antenna have been studied in detail [22,23]. The advantage of the magnetic dipole antenna is that it provides lower impedance characteristics, corresponding to those of human tissues. The use of a magnetic dipole is advantageous in terms of achieving characteristics that best match those inside the human body. In order to establish a stable wireless link, the wireless link needs to be estimated using an efficient method, including the selection of the optimal antenna to provide the best impedance characteristics inside the human body.

**Figure 1:**
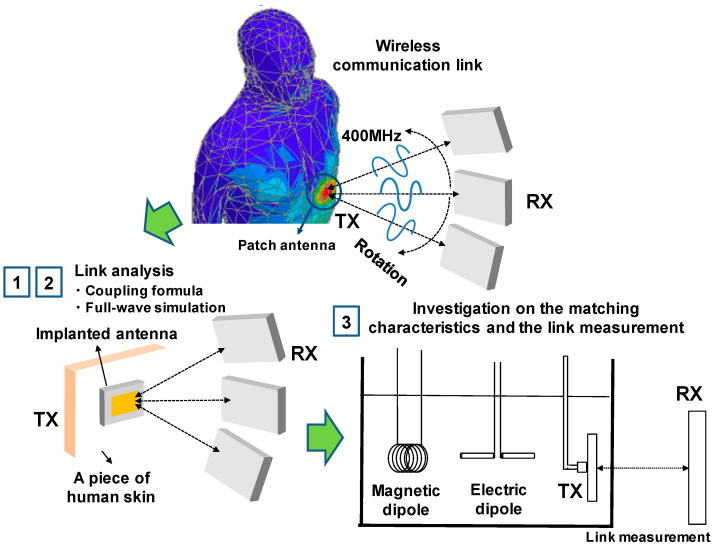
Overview of this research study for the evaluation of the communication link between two antennas.

In this paper, radio transmission between antennas that are placed inside and outside the human body is studied in the context of realizing a stable wireless communication link. The wireless link is used to transfer pacemaker data, which operates at 402–405 MHz for a medical implant communication system (MICS). Figure 1 depicts the evaluation scheme for radio transmission between antennas inside and outside the human body. The complete wireless communication link is evaluated in terms of the efficient estimation of the wireless link and the best matching characteristics inside the human body. The key milestones of this work are that: (1) The coupling formula is applied to estimate the wireless link for biomedical applications; (2) for use in biomedical applications, the formula is slightly modified in terms of including characterization of the far-field pattern inside the body and adding a reflection coefficient term between the implanted antenna and the human body; (3) impedance matching in the environment of the human body was studied through representative examples, such as electrical and magnetic dipole antennas. The wireless link was computed using the coupling formula and the result was compared with full-wave simulation, FEKO, and measurements. The wireless link was measured using phantom fluid that provides relative permittivity $\varepsilon_{r}$ = 46.4 and conductivity σ = 0.67, which is similar to the characteristics of the human skin. The measured result agrees well with the computed result and FEKO simulations.

## 2. Computation of the Antenna Coupling in the Near-Field and Far-Field Region

The power transmission is computed using the integral coupling formula based on the 3D vector far-field pattern. The formula computes the antenna coupling in the near-field and far-field region. The formula was modified slightly to provide an accurate link analysis for biomedical applications. The far-field pattern of the antenna placed inside human body was characterized using a piece of human skin, with the associated antenna. Furthermore, the impedance mismatch of the antenna in the human body environment was added in order to include the reflection coefficient between the antenna and the human body. The formula was used to evaluate the link between two biomedical antennas placed in various configurations.

### 2.1. Revisiting the Integral Coupling Formula

**Figure 2:**
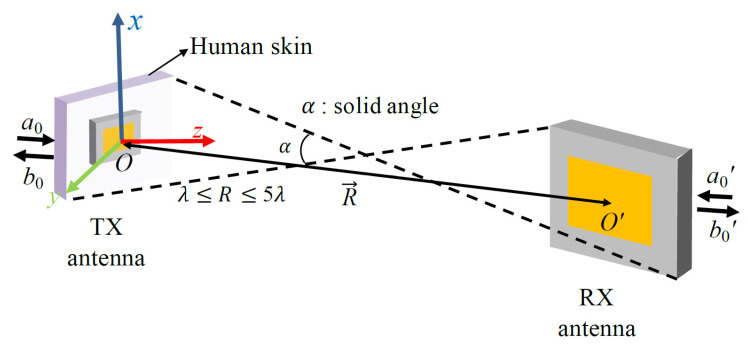
Geometries of the mutual coupling between the two antennas used for biomedical applications.

The transmission integral with the plane–wave scattering matrix (PWSM) theory has expanded its applicability to arbitrarily oriented antennas [15]. The coupling formula in [16,18], based on a normalized vector far-field pattern, was derived from the transmission integral. The formula utilizes the complex vector far-field pattern, which is easily attainable through numerical methods or measurements. In this section, the essentials of the coupling formula presented in [16,18] are revisited. The geometries of the two arbitrarily positioned antennas are shown in Figure 2. The coupling quotient can be determined based on the relationship between the amplitude of the input wave of the transmitting antenna, $a_{0}$, and the output wave of the receiving antenna, $b_{0}^{\prime }$

(1) $$\frac{{b^{\prime }}_{0}}{a_{0}}\left(\vec{R}\right)=-\frac{C}{4\pi k}\iint_{\sqrt{\left(k_{x}^{2}+k_{y}^{2}\right)\;}<\;k}^{}\frac{{\vec{f}}_{TX}\left(\vec{k}\right)\cdot {\vec{g}}_{RX}\left(-\vec{k}\right)}{k_{z}}\;e^{i\vec{k}\cdot \vec{R}}\;dk_{x}\;dk_{y}$$

where $\vec{k}=k_{x}\hat{x}+k_{y}\hat{y}+k_{z}\hat{z}$ is the wave vector in free space and ${\vec{f}}_{TX}\left(\vec{k}\right)$ and ${\vec{g}}_{RX}\left(-\vec{k}\right)$ are the complex vector far-field pattern of the transmitting and receiving antenna, respectively. A double integral of the scalar product between the two vector far-field patterns was used to calculate the coupling quotient. The relationship between the coupling quotient and the S_21_ can be defined as $S_{21}={\left|b_{0}^{\prime }/a_{0}\right|}^{2}$.

Reductions in the integration range, such as $\sqrt{\left(k_{x}^{2}+k_{y}^{2}\right)}<\;k$, only provides propagating waves, while it neglects evanescent waves. It is worth noting that Formula (1) depends on the $e^{-i\omega t}$ time convention and neglects multiple reflections. The solid angle *α* can be defined as the angle subtended by two spheres enclosing the transmitting and receiving antennas. The first step is the acquisition of the 3D vector far-field patterns of the two antennas. The 3D vector far-field pattern can be acquired through numerical methods, full-wave simulations, and measurements. One must obtain the far-field pattern at the phase center of the antenna. The phase center of the antenna is placed at the point where the uniform phase response of the far-field pattern is observed. This procedure will be helpful in terms of achieving sufficient convergence of the calculation. In this study, the far-field pattern characterized in the human body environment was employed. It was demonstrated in [8], that the use of a small piece of human skin creates similar EM characteristics to those of the entire human body. Therefore, the antenna was implanted inside a small piece of human skin to characterize the far-field pattern. The next step is to perform the coordinate transformation in order to create the geometries of the two antennas. Eulerian angles were used to transform the coordinate system of each antenna into the global coordinate system. The relative orientation and separation distance between two antennas can be created using coordinate transformation. An interpolation process was used to evaluate the far-field pattern at the sampling points on the *x*–*y* plane.

**Figure 3:**
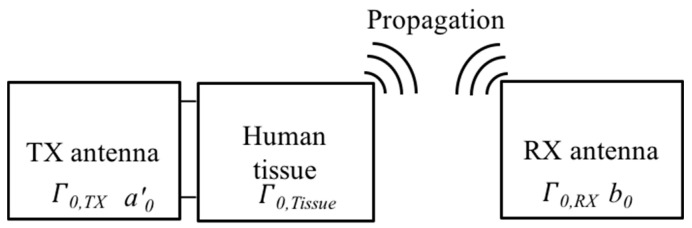
Description of the two-port network for the wireless communication link between the transmitting implanted antenna placed inside the human skin and the receiving antenna outside the human tissue.

The impedance matching of the antenna placed inside and outside human body represents an important consideration in realizing a stable communication link. Therefore, it is advantageous to contain the reflection coefficient between an antenna and the human body. Figure 3 shows the two-port network for the impedance mismatch term *C*. It was assumed that the transmitting antenna is placed inside the human skin, as discussed in Figure 1. The complete impedance matching term, constant *C*, can be expressed as

(2) $$C=\frac{Z_{FL,RX}}{Z_{0}}\frac{1}{\left(1-\Gamma_{0,\;TX}\Gamma_{0,\;Tissue}\right)}\frac{1}{\left(1-\Gamma_{0,\;RX}\right)}$$

where $Z_{FL,RX}$ is the impedance of the receiving antenna feedline and $Z_{0}$ is the intrinsic impedance in a free space. For the transmitter, *Γ_0,TX_*, and *Γ_0,Tissue_* indicate the reflection coefficient of the transmitting antenna and human tissue, respectively. For the receiver, *Γ_0,RX_*, represents the reflection coefficient of the receiving antenna. It was assumed that the transmitting and the receiving antenna are perfectly matched to the source and the load, respectively. The detailed procedure to derive the impedance matching term *C* is provided in Appendix A.

The computer program in [18] was developed in order to compute the antenna coupling between two antennas, which allows for flexibility in terms of the relative axis, orientation, and arbitrary antenna movement. Furthermore, the sampling frequency has been introduced to adjust the size of the integration for sufficient convergence. The sampling frequency presented in [18] is

(3) $$f_{s}=2\kappa \times \left(D_{TX}+D_{RX}\right)$$

where *D_Tx_* and *D_Rx_* are the diameters of the transmitting and the receiving antennas, respectively, and κ is termed the oversampling ratio. One can use the constant *κ* in order to change the sampling frequency. Spectrum integration is confined to the solid angle *α* subtended by the diameter *D_Tx_* and *D_Rx_*. This confinement is an important requirement in reducing the necessary computational resources. The recent advances in computer capabilities have allowed for the testing of the converged results using different solid angles. It was demonstrated in [19] that the utilization of a larger solid angle *α* is beneficial for offering a degree of improvement in terms of the converged results. The upper and lower bounds for the effective separation distance can be defined as

(4) $$\frac{D_{TX}+D_{RX}}{2}<R<\frac{{\left(D_{TX}+D_{RX}\right)}^{2}}{\lambda }$$

The integral form of coupling formula was implemented using the double summation at the sampling points $(k_{x}$-$k_{y}$). The summation form of the formula makes it possible to implement through the computer program presented in [18]. The summation form of the formula is presented as

(5) $$\frac{{b^{\prime }}_{0}}{a_{0}}\left(\vec{R}\right)=-\frac{C}{k}{\left(\Delta k\right)}^{2}\times \underset{m}{\sum }\underset{n}{\sum }\frac{{\vec{f}}_{TX}\left(k_{x}^{mn},\;k_{y}^{mn}\right)\cdot {\vec{g}}_{RX}\left(k_{x}^{mn},\;k_{y}^{mn}\right)}{k_{z}^{mn}}\;\times e^{i{\vec{k}}^{mn}\cdot \vec{R}}$$

where $k=\hat{x\;}k_{x}^{mn}+\hat{y\;}k_{y}^{mn}+\hat{z\;}k_{z}^{mn}$ and $\Delta k=\frac{2\pi }{f_{s}}$ = $\frac{\pi }{\kappa \left(D_{TX}+D_{RX}\right)}$.

The index *m*, *n* for the double summation can be defined as

(6) $$1\leq m,\;n\leq \frac{2{\left(D_{TX}+D_{RX}\right)}^{2}}{\lambda R}$$

Note that *κ* = 4 is used for the index *m*, *n*. The upper bound of the effective range is restricted to compute the antenna coupling in the far-field region. The complete form of the Friis formula, including the reflection coefficient between the implanted antenna and the human skin, can be defined as

(7) $${\left|\frac{{b^{\prime }}_{0}}{a_{0}}\right|}^{2}={\left(\frac{\lambda }{4\pi R}\right)}^{2}G_{TX}\left(\theta ,\phi \right)G_{RX}\left(\theta ,\phi \right)\times {\left|{\hat{\rho }}_{TX}\cdot {\hat{\rho }}_{RX}\right|}^{2}\left(1–{\left|\Gamma_{0,\;TX}\Gamma_{0,Tissue\;}\right|}^{2}\right)\left(1–{\left|\Gamma_{0,\;RX}\right|}^{2}\right)$$

where $G_{TX}\left(\theta ,\phi \right)$ and $G_{RX}\left(\theta ,\phi \right)$ represent the far-field gain of the transmitting antenna and the receiving antenna, respectively, and ${\hat{\rho }}_{TX}$ and ${\hat{\rho }}_{RX}$ are the polarization vectors of the transmitting antenna and the receiving antenna, respectively. It was assumed that both the transmitting and receiving antennas are connected to the identical feed line, and that there was no reflection from the source and the load.

### 2.2. Electromagnetic Characterization of Implanted Antennas

In order to establish a reliable communication link, implanted antennas need to be investigated in terms of impedance matching the characteristics present inside the human body. The characteristics of the wave impedance vary according to the different kinds of antennas. The human body typically has a large dielectric constant, causing low impedance characteristics. Therefore, the use of an antenna with lower wave impedance is beneficial in order to provide the best matching characteristics.

**Figure 4:**
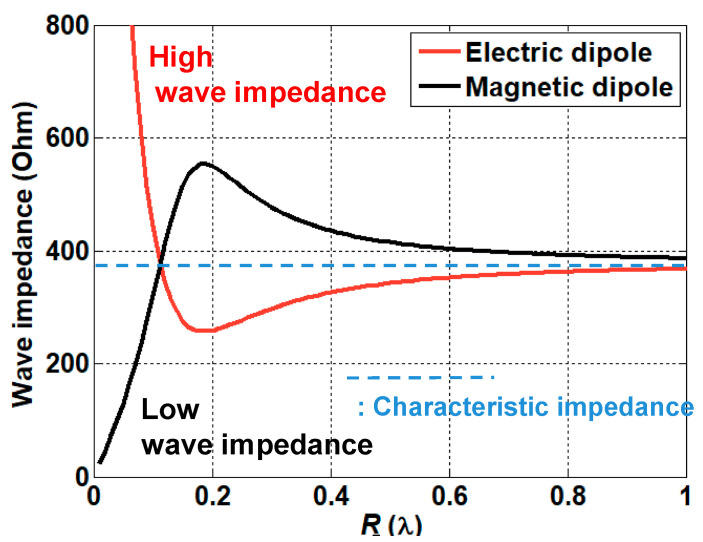
Wave impedance of the electric dipole and the magnetic dipole antenna.

The wave impedance of the electric dipole $Z_{E}\left(r\right)$ and the one of the magnetic dipole $Z_{H}\left(r\right)$ can be derived from the ratio between the electric and magnetic field of each antenna [22,23], given by

(8) $$Z_{E}\left(r\right)=\eta_{0}\left|\frac{\left[1+\frac{1}{j\beta r}+\frac{1}{{\left(j\beta r\right)}^{2}}\right]}{\left(1+\frac{1}{j\beta r}\right)}\right|$$

(9) $$Z_{H}\left(r\right)=\eta_{0}\left|\frac{\left(1+\frac{1}{j\beta r}\right)}{\left[1+\frac{1}{j\beta r}+\frac{1}{{\left(j\beta r\right)}^{2}}\right]}\right|$$

where $\eta_{0}$ and β represent the characteristic impedance of the free space and the propagation constant of the free space, respectively. Figure 4 shows the comparison of the wave impedance of the electric dipole and the magnetic dipole. As previously discussed, the magnetic dipole provides low impedance characteristics, while the electric dipole shows an opposite trend in terms of impedance characteristics. The impedance inside the human body can be derived from taking into account the different kinds of electrical properties of the human body using the relationship

(10) $$Z=\;\sqrt{\frac{\mu }{\varepsilon }}=\sqrt{\frac{1}{\varepsilon_{r}}}\sqrt{\frac{\mu_{0}}{\varepsilon_{0}}}=\sqrt{\frac{1}{\varepsilon_{r}}}\eta_{0}$$

where $\mu_{0}$ and $\varepsilon_{0}$ are the free space permeability and permittivity, respectively, and $\varepsilon_{r}$ is the relative permittivity which can be determined by the electrical properties of the human tissue. The reflection coefficient between antenna and human tissue can be derived using

(11) $$\Gamma_{0,\;Tissue}=\frac{Z_{A}-Z_{Tissue}}{Z_{A}+Z_{Tissue}}$$

where *Z_A_* and *Z_Tissue_* are the impedance of the antenna and the human tissue, respectively.

## 3. Results

In this section, the complete wireless communication link is investigated including calculation of the wireless link and analysis of the matching characteristics inside and outside the human body. It was assumed that the TX antenna is located inside the skin, while the RX antenna is placed outside the human body to establish communication links for the pacemaker system. All evaluations were performed at the center frequency of 400 MHz.

### 3.1. Calculation of the Link between Two Antennas

The coupling formula was used to evaluate the link between the two antennas. This section contains the evaluations for: (1) the free space link, and (2) the link between the two antennas placed inside and outside the human body. The computed results are compared with the results obtained using a full-wave simulation FEKO. The calculated results show an excellent level of agreement with the results from the full-wave simulation FEKO.

#### 3.1.1. Antenna Design Used for the Link Analysis

**Figure 5:**
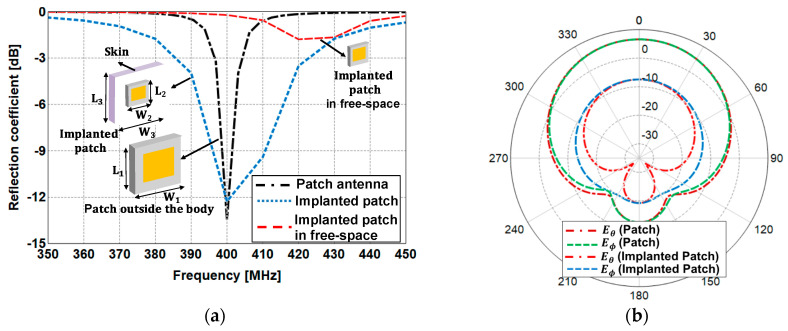
(**a**) Impedance matching characteristics of the patch antennas used for the link analysis and (**b**) the radiation patterns of the patch antennas at 400 MHz.

The microstrip patch antennas are designed to operate inside and outside the human body. The size of the patch antenna outside the human body was (L_1_ × W_1_) = (40 cm × 40 cm), while the patch antenna inside the human body was smaller at (L_2_ × W_2_) = (20 cm × 20 cm) in order to provide the best matching characteristics inside the human body. The patch antenna outside the human body was designed on a 3.2 mm thick substrate with $\varepsilon_{r}$ = 2.2 tanδ (loss tangent) = 0.0009. For the patch antenna inside the human body, the thickness of the substrate was set as 6.4 mm and the microstrip antenna was printed in the middle of the substrate with $\varepsilon_{r}$ = 10.2 and tanδ = 0.0025. The size of the skin tissue was set as (L_3_ × W_3_) = (25 cm × 25 cm), with a thickness of 4 mm above the patch antenna. The characteristics of impedance matching for the patch antennas are shown in Figure 5a. The patch antennas placed inside and outside the human body provide reasonably good matching characteristics of less than −10 dB around 400 MHz, however, the characteristics of the implanted patch deteriorate when it operates in air. Figure 5b shows a comparison of the radiation patterns in diverse environments. The patch antenna placed outside the human body has a maximum gain of 6.8 dB, while the other one placed inside the human body has a maximum gain of −7.65 dB. The patch antennas were used to evaluate the free-space link and the link between the implanted antenna and the outside receiving antenna.

#### 3.1.2. Free-Space Link Analysis

Prior to estimating the link, including the implanted antenna, the link was evaluated using the two microstrip patch antennas placed in a free space. The coupling formula was used to compute the coupling versus the separation distance and the coupling versus the transverse displacement [16,17,18]. In this section, the coupling formula is applied to both of the antenna displacements.

**Figure 6:**
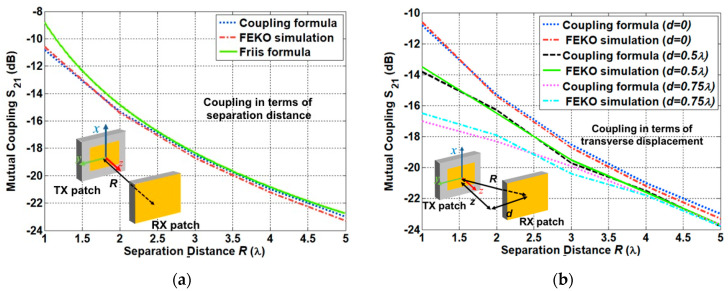
Free-space link analysis for (**a**) the coupling in terms of separation distance normalized with the wavelength at 400 MHz and (**b**) the coupling in terms of transverse displacement normalized with the wavelength at 400 MHz.

For the coupling versus separation distance, the antenna coupling was evaluated by moving the RX patch antenna in terms of the *R* direction. For the coupling versus transverse displacement, the receiving antenna was located at different transverse displacements, and the antenna coupling was evaluated. The antenna coupling for the first scheme is shown in Figure 6a. It is shown that the coupling formula provides the coupling level which is roughly 1.5 dB lower than the one of the Friis formula in the closest distance. This is attributed to the gain reduction effect in the near-field region. The computed results show a good agreement with the full-wave simulation FEKO with a deviation less than 0.6 dB. The second scheme was evaluated as shown in Figure 6b. The coupling was evaluated for the transverse displacements of 0.5 and 0.75 λ. There is a significant difference in the coupling level for different transverse displacements in close proximity, while all curves converge to a coupling level in the far-field region. This is because the offset geometry in close proximity is more influential in determining mutual coupling. There was a 1 dB deviation between the computed and the simulated results.

#### 3.1.3. Link between the Two Antennas Inside and Outside the Human Body

**Figure 7:**
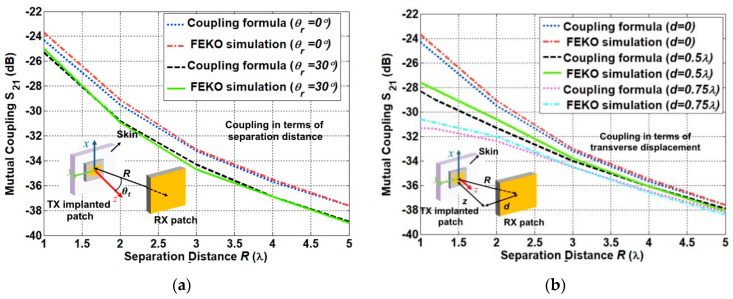
Link between the two antennas inside and outside the human body for (**a**) the coupling in terms of separation distance, normalized with the wavelength at 400 MHz and (**b**) the coupling in terms of transverse displacement, normalized with the wavelength at 400 MHz.

The link between two patch antennas placed inside and outside the human body was evaluated. The patch antenna embedded in a piece of human tissue was used to represent the implanted antenna inside the human skin. It was reported in [8], that the simplified model provides similar characteristics to those of the antenna implanted inside the large human body model. This will help reduce the required computational resources. The attenuation due to the propagation inside the human body is simply characterized through utilizing the difference between the maximum antenna gain of the implanted transmitting patch and that of the receiving patch. The antenna coupling was evaluated for the first scheme with the inclusion of a rotational one, as shown in Figure 7a. For an accurate estimation, the rotation angles are restricted to the angles which are smaller than $\theta_{t}$ = 30° [17]. Therefore, the coupling curves were obtained at the rotation angles $\theta_{t}$ = 0° and $\theta_{t}$ = 30°. It can be observed that the deviation between the computed and simulated results is less than 0.8 dB for both scenarios. The difference between the two coupling curves agrees well with the one of the radiation pattern at the different rotation angles. Coupling curves for the second scheme are shown in Figure 7b. The deviation between the computed and simulated results is 1.2 dB, which is slightly higher than that of the free-space link.

### 3.2. Matching Characteristics Inside and Outside the Human Body

**Figure 8:**
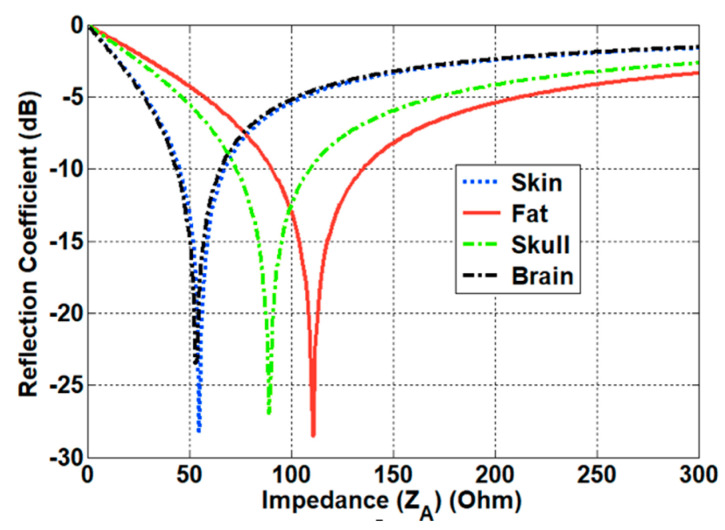
Matching characteristics of an antenna inside the different parts of the human body.

**Figure 9:**
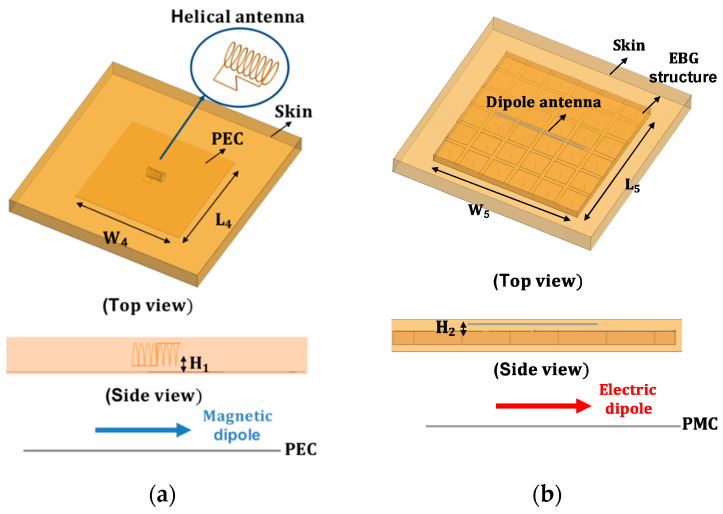
Configuration of the representative antennas: (**a**) helical antenna mounted on PEC and (**b**) dipole antenna mounted on EBG structure.

The impedance matching inside the human body was investigated based on the characteristics of the implanted antennas. Owing to the electrical properties of the human body, the antenna design needs to compensate for the differences between free space and the human body. To investigate the characteristics of the antenna inside the body, we evaluated the design of a helix antenna on PEC (Perfect Electric Conductor) ground and a dipole antenna mounted on an EBG (Electromagnetic Band Gap) structure, which are representative examples of magnetic and electric dipole antennas, respectively. In particular, a small helix antenna is favorable—due to its miniaturized design—for implantation inside the human body. The wave impedance of the representative antennas was obtained using a full-wave simulation FEKO. The simulated results were compared with the theoretical results discussed in Section 2.2. As expected, the curve of the helix antenna resembles that of the magnetic dipole, while the curve of the dipole antenna is similar to that of the electric dipole. In particular, the helix antenna provides low impedance characteristics in close proximity. This corresponds to the characteristics of the magnetic dipole, and this antenna will possess optimal values that are analogous to the human body. Using Equation (10), the impedances of different parts of the body were calculated. The computed results are provided in Table 2. It can be seen that the impedance of the human body is lower than the characteristic impedance of the free space. Based on the impedance of the skin and the wave impedance of the antenna, the matching characteristics were acquired from Equation (11). Figure 8 shows the reflection coefficient in terms of the different wave impedance of the antenna. The best matching characteristics are obtained when the impedance of the antenna is similar to that of the tissue. The matching characteristics were evaluated through using representative antennas, such as magnetic dipole and electric dipole antennas. A small helical antenna on the PEC was selected as an example of the magnetic dipole antenna, while a dipole antenna on the EBG structure was chosen as an example of the electric dipole antenna. The configuration of the representative antennas is shown in Figure 9. For the helical antenna, the radius and the height were set as 2.1 and 9 mm, and the height from the PEC to the center of the helical antenna (H_1_) was designed as 3.4 mm. The size of the PEC was set as (L_4_ × W_4_) = (5.4 cm × 5.4 cm). The dipole-EBG was designed and scaled based on a previous study [24]. One difference is that the miniaturized 6 × 6 EBG structure was used in this study. The size of the EBG structure was set as (L_5_ × W_5_) = (9.7 cm × 9.7 cm).

**Figure 10:**
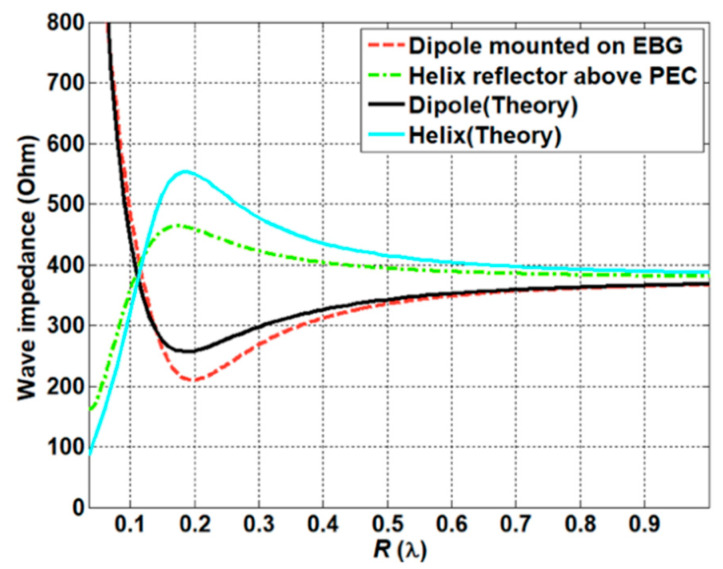
Wave impedance of the dipole on EBG and helix antenna on PEC, and a comparison to the calculated wave impedances of the dipole and the helix antenna.

**Figure 11:**
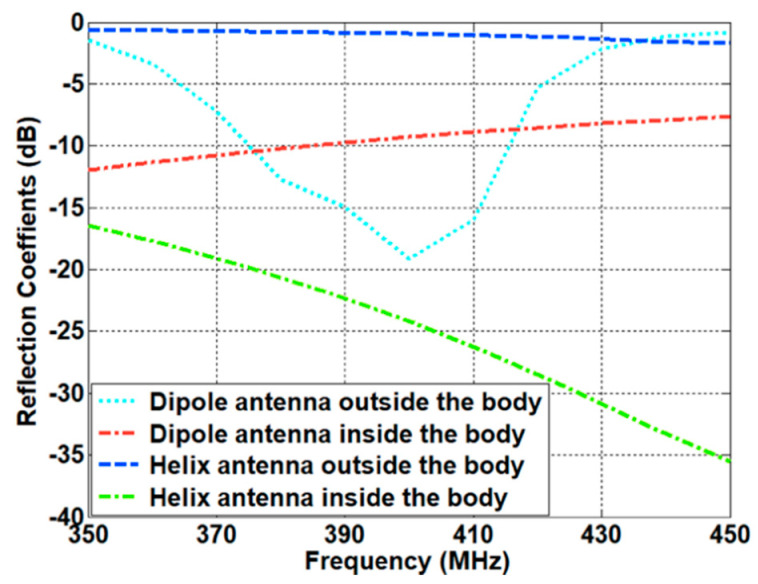
Simulated impedance matching characteristics inside and outside the body (the skin tissue).

The height from the EBG structure to dipole antenna (H_2_) was set as 2.2 mm. The helical antenna on the PEC corresponds to the magnetic dipole on the PEC, while the dipole antenna on the EBG structure represents the electric dipole on the PMC. The wave impedance of the two antennas was investigated in order to evaluate the matching characteristics inside the human body. Figure 10 shows the simulated results of the wave impedance and the calculated wave impedance of the electric and magnetic dipole antenna. It can be seen that the wave impedance of the helical antenna is similar to that of the magnetic dipole antenna, while the one of the dipole antenna resembles the trend of the electric dipole antenna. In particular, within close proximity, the magnetic dipole possesses low impedance, and the electric dipole has high impedance, when compared to the characteristics of impedance in air. The matching characteristics of the two kinds of antennas were investigated by placing the antennas inside the human body (the skin tissue). Figure 11 shows the simulated impedance characteristics inside and outside the skin tissue. As predicted, the helical antenna provides the best matching characteristics inside the skin tissue, while it shows the deteriorated one outside the skin tissue. In contrast, the electric dipole exhibits slightly degraded matching characteristics inside the skin tissue when compared to those outside the body. It was demonstrated that the magnetic dipole type antenna is advantageous for providing the best matching characteristics inside the human body.

## 4. Measurements

**Figure 12:**
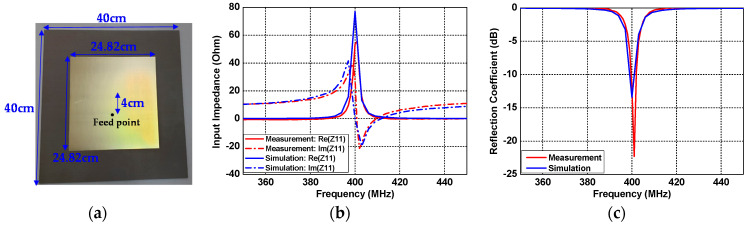
Patch antenna outside the human body: (**a**) fabricated structure, (**b**) input impedance Z_11_, and (**c**) reflection coefficient S_11_.

Experiments were conducted to verify the calculated and simulated results of the wireless link. After the experiment to verify the impedance matching performance of the patch antenna inside and outside the human body, the coupling between the antennas was measured by changing the separation distance. The MS46522B model vector network analyzer(VNA) from the company, Anritsu, was used for the experiment. Figure 12a shows the patch antenna outside the human body, fabricated for operating at a frequency of 400 MHz. The patch antenna was fabricated on a Taconic TLY-5 substrate (*ε_r_* = 2.2, tan*δ* = 0.0009) with a thickness of 3.2 mm, and the size of the ground and the conducting patch were 40 cm × 40 cm and 24.82 cm × 24.82 cm, respectively. The reader patch was fed from the SubMiniature version A(SMA) connector from the company, WithWave, and the feed position was located 4 cm from the patch center to improve the impedance matching. The fabricated implant patch antenna operates with the performance of Z_11_ = 55.7 − *j*7.07 Ω and S_11_ = −22.3 dB at 401 MHz, which is the result of a 1 MHz up shift compared to the center frequency of the simulated result. Nevertheless, the patch antenna outside the human body operates with excellent performance with Z_11_ = 53.5 + *j*28.7 Ω and S_11_ = −12 dB at 400 MHz, and the impedance matching characteristic matches well with the simulated result, as shown in Figure 12b,c.

**Figure 13:**
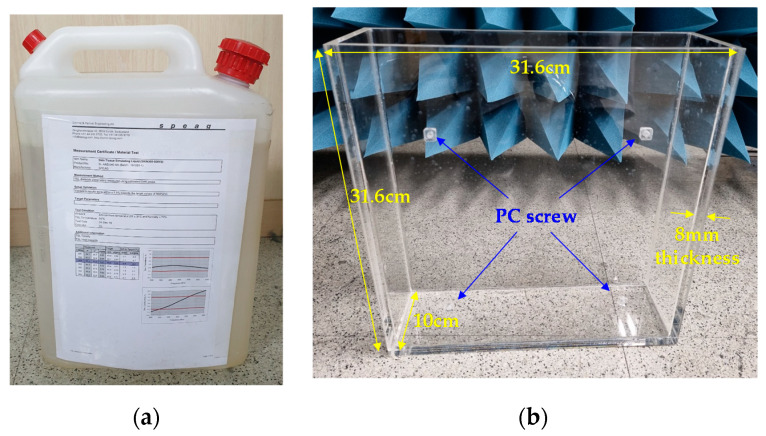
Implementation of the human body (**a**) skin tissue liquid and (**b**) acrylic tank.

The measurement of the implant patch antenna was conducted both in skin tissue liquid (corresponding to inside the human body) and in free-space (corresponding to outside the human body). For the human body, 10 L of Skin Tissue Simulating Liquid (SKIN350-500V2) from Schmid & Partner Engineering AG company (Zürich, Switzerland) was used as shown in Figure 13a. The relative permittivity and conductivity values are *ε_r_* = 46.4 and *σ* = 0.67 at room temperature (22 °C) and a frequency of 400 MHz, which are specified in the data sheet. Figure 13b shows the 10 L acrylic tank used to contain the skin tissue liquid. The size of the acrylic tank was made to be 31.6 cm × 31.6 cm × 10 cm considering the size of the implant patch antenna, and the wall thickness was set to 8 mm, considering the relative density of the skin tissue liquid (1.2–1.4 kg/L). It is worth noting that the 8mm of the wall thickness corresponds to about 0.01 λ, so its effect on the radiation performance of the implant patch antenna is negligible. The antenna was assumed to be located 4 mm from the surface of the acrylic tank, and M5 size polycarbonate (PC) screw hole structures were added to stably mount the implant patch.

**Figure 14:**
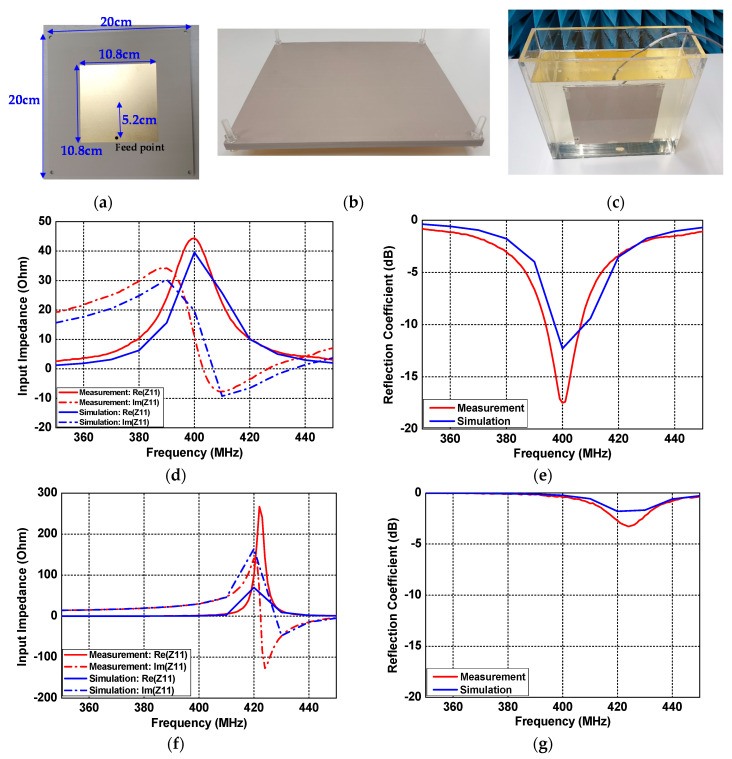
Patch antenna inside the human body: (**a**) lower layer of fabricated structure, (**b**) assembly of the fabricated structure, (**c**) antenna measurement setup in skin tissue liquid, (**d**) input impedance Z_11_ in skin tissue liquid, (**e**) reflection coefficient S_11_ in skin tissue liquid, (**f**) input impedance Z_11_ in free space, and (**g**) reflection coefficient S_11_ in free space.

The patch antenna inside the human body consisted of a dielectric substrate (metal free) on the upper layer and a conducting patch with a grounded dielectric slab on the lower layer. Figure 14a shows the lower layer of the patch antenna fabricated for an operating frequency of 400 MHz inside the skin tissue. The patch antenna was designed on Taconic RF-10 substrate (*ε_r_* = 10.2, tan*δ* = 0.0025) with a thickness of 3.2 mm, and the sizes of the ground and the conducting patch were 20 cm × 20 cm and 10.8 cm × 10.8 cm, respectively. The feed position of the implant patch was located 5.2 cm from the patch center to improve the impedance matching. Figure 14b shows the structure in which the upper layer and the lower layer were assembled. Both layers were assembled with M5 size PC screws, and were mounted through screw holes in the acrylic tank. To prevent the occurrence of air gaps and leakage of skin tissue, waterproof tape was firmly attached to the four corners of the assembed structure. Finally, the assembled structure was fixed by screwing into an acrylic tank containing skin tissue liquid, as shown in Figure 14c. Figure 14d–g shows the measured results of the fabricated implant patch antenna. Similar to the simulation, the implant patch antenna operated at 400 MHz in the skin tissue, and operated near 420 MHz in the air, due to the decrease in relative permittivity. The measured input impedance and reflection coefficients were Z_11_ = 44.4 + *j*11.2 Ω and S_11_ = −17.5 dB at a 400 MHz frequency in the skin tissue, as shown in Figure 14d and e. The frequency downshift occurred in the measured results due to a small air gap by soldering the SMA connector.

**Figure 15:**
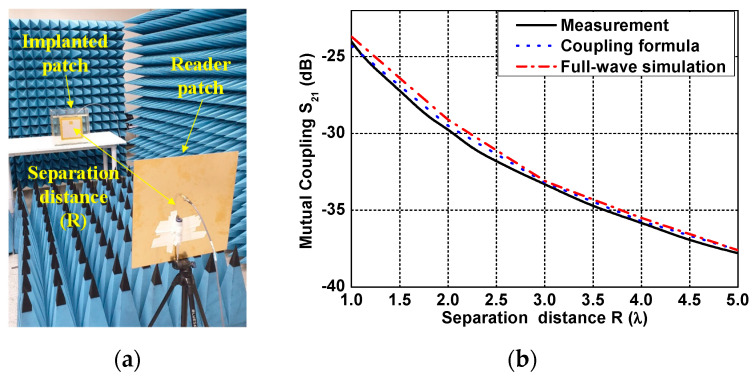
Measurement of coupling between the reader patch and the implant patch antennas (**a**) measurement setup and (**b**) measured result.

The measurements were conducted to verify the coupling formula using the fabricated patch antennas. Figure 15a shows the measurement setup of the coupling between one patch antenna inside the phantom fluid tank and the other one in air. The two antennas were set to face each other in the broadside direction, and the coupling was measured from S_21_ of the VNA as a separation distance R changes from 0.5–5λ (correspond to 37.5–375 cm). The measured coupling S_21_ shows a similar tendency to the results calculated from the coupling formula and full-wave simulation, as shown in Figure 15b. The ground effect caused by the floor generated at the far separation distance was minimized by the installed microwave absorber. However, at a near separation distance, a measurement error of 0.4 dB occurred due to the reflected wave from the table used in the measurement.

## 5. Discussion

The coupling formula is advantageous since it enables us to compute the near-field antenna coupling based on the far-field radiation pattern. The coupling formula was applied to compute the wireless link between two antennas inside and outside of the human body. The patch antenna inside the small part of the human tissue was used to characterize the far-field pattern of the implanted antenna. The computed results obtained from the coupling formula show good agreement with the full-wave simulation FEKO and measurements. The deviation between the computed results and full-wave simulation was less than 0.6–1.2 dB for both cases versus separation distance and transverse displacement. The matching characteristics inside the human body was investigated in terms of the electric and magnetic dipole antennas. The representative examples of the electric and magnetic dipole antennas were selected as the dipole antenna on the EBG structure and the helical antenna on the metal ground plane, respectively. It was found that the magnetic dipole provides low impedance characteristics which are similar to those of the human body, which is advantageous in terms of providing optimal impedance matching inside the human body. The indoor measurement was performed using one patch antenna inside the phantom fluid and the other one in air. The measured results show a good level of agreement with the simulated results in terms of matching characteristics and link performance. This study provides an important guideline for the creation of reliable wireless links based on an accurate numerical method and antenna design in terms of matching characteristics.

## Figures and Tables

**Table 1:** Comparison among different methods for estimating the wireless link.

Methods	Effective Range	Accuracy	Computational Complexity
Inductive coupling [5,6,7]	Non-radiative reactive near-field	High	High
Radiative Mid-field [10,11]	Radiative near-field and far-field region	Low	High
Coupling formula [16,17,18,19,20]	Radiative near-field and far-field region	High	Low

**Table 2:** Impedances of the different parts of the human body.

Tissue	$\mathrm{Relative}\;\mathrm{Permittivity}\;(\varepsilon_{r})$	Impedance (Z*_Tissue_*)
Skin	46.7	55.2 Ω
Fat	11.6	110.7 Ω
Skull	17.8	89.4 Ω
Brain	49.7	53.5 Ω

**Figure A1:**
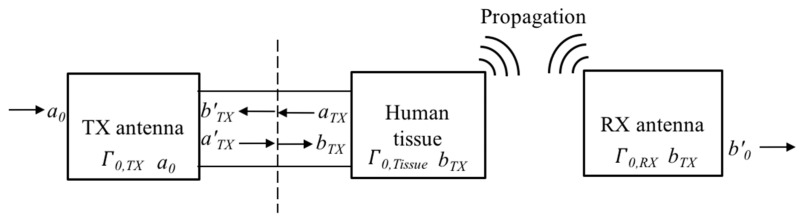
Description of the two-port network used for the derivation of the impedance mismatching term.

## Data Availability

The data presented in this study are available on request from the corresponding author.

## Appendix A

The impedance mismatch of an antenna inside the human body was proposed in order to estimate an accurate coupling quotient. In this Appendix, the derivation of the antenna mismatch term is presented in detail. Figure A1 shows the two-port network, linked between the transmitting antenna and the receiving antenna. Except for the propagation part, the coupling quotient between the transmitting antenna and the receiving antenna is derived, which is identical to the impedance mismatching term. One can start with the mismatch term of transmitting antenna. The mismatch term between the TX antenna and the human tissue can be defined as

(A1) $$a_{TX}=\Gamma_{0,\;Tissue\;}b_{TX}$$

(A2) $$a_{TX}^{\prime }=\Gamma_{0,\;TX\;}b_{TX}^{\prime }+a_{0}$$

Substituting Equation (A1) into Equation (A2) based on the relationship $a_{TX}^{\prime }$ = $b_{TX}$ and $a_{TX}$ = $b_{TX}^{\prime }$ will produce

(A3) $$b_{TX}=\frac{a_{0}}{1-\Gamma_{0,\;TX\;}\Gamma_{0,\;Tissue\;}}$$

For the receiving antenna, the amplitude of the received wave can be derived using the following relationship

(A4) $$b_{TX}-b_{TX}\Gamma_{0,\;RX}=b_{0}^{\prime }$$

Substituting Equation (A3) into Equation (A4), the coupling quotient can be derived as

(A5) $$\frac{b_{0}^{\prime }}{a_{0}}=\left(\frac{1}{1-\Gamma_{0,\;Tissue\;}}\right)\frac{1}{1-\Gamma_{0,\;RX}}$$

It was assumed that both the transmitting and receiving antennas are fed by an identical waveguide. Note that multiple reflection is ignored in the derivation. The Equation (A5) was applied to compute the coupling quotient presented in Equation (1).
